# Chasing the mechanisms of ecologically adaptive salinity tolerance

**DOI:** 10.1016/j.xplc.2023.100571

**Published:** 2023-03-07

**Authors:** Silvia Busoms, Sina Fischer, Levi Yant

**Affiliations:** 1 Plant Physiology Laboratory, Bioscience Faculty, Universitat Autònoma de Barcelona, Bellaterra, Barcelona E-08193, Spain; 2 Future Food Beacon of Excellence, University of Nottingham, Nottingham NG7 2RD, UK; 3 School of Biosciences, University of Nottingham, Nottingham NG7 2RD, UK; 4 School of Life Sciences, University of Nottingham, Nottingham NG7 2RD, UK

**Keywords:** adaptation, salinity, polyploidy, microbiome, evolution, ecology

## Abstract

Plants adapted to challenging environments offer fascinating models of evolutionary change. Importantly, they also give information to meet our pressing need to develop resilient, low-input crops. With mounting environmental fluctuation—including temperature, rainfall, and soil salinity and degradation—this is more urgent than ever. Happily, solutions are hiding in plain sight: the adaptive mechanisms from natural adapted populations, once understood, can then be leveraged. Much recent insight has come from the study of salinity, a widespread factor limiting productivity, with estimates of 20% of all cultivated lands affected. This is an expanding problem, given increasing climate volatility, rising sea levels, and poor irrigation practices. We therefore highlight recent benchmark studies of ecologically adaptive salt tolerance in plants, assessing macro- and microevolutionary mechanisms, and the recently recognized role of ploidy and the microbiome on salinity adaptation. We synthesize insight specifically on naturally evolved adaptive salt-tolerance mechanisms, as these works move substantially beyond traditional mutant or knockout studies, to show how evolution can nimbly “tweak” plant physiology to optimize function. We then point to future directions to advance this field that intersect evolutionary biology, abiotic-stress tolerance, breeding, and molecular plant physiology.

## Teaser

Adaptive natural responses to saline soils serve as powerful examples of evolutionary change and allow inference for rational crop development. Mechanistic insight into these evolved responses has increased dramatically, with notable progress in our understanding of the effects of polyploidy and the microbiome. This review synthesizes these works, highlighting benchmark studies deconstructing mechanisms of adaptation using genomic, functional, and ecological approaches.

## Widespread, but costly, and transitory? The evolution of salinity tolerance

While sodium is an essential plant nutrient, high concentrations of Na^+^ ions severely inhibit growth (Bernstein, 1975; Greenway and Munns, 1980). This effect, termed salinity stress, is linked to water uptake challenges (Reina-Sánchez et al., 2005), impaired metabolic processes (Che-Othman et al., 2017), and decreased photosynthesis (Ashraf and Harris, 2013). Plants can employ diverse strategies to mitigate these impacts, with the result that rapid adaptive evolution is seen in many taxa, mediating varying degrees of tolerance. At the high end, the term halophyte is reserved for lineages endemic to salty habitats, specifically growing in salinities greater than 200 mM NaCl (Flowers and Colmer, 2008).

Salinity tolerance can arise rapidly and can vary dramatically between species (Flowers et al., 2010). This rapid evolution has been linked to dynamic environmental conditions that serve as drivers of plant adaptation to salinity and other soil-related (edaphic) stressors (Cheeseman, 2015). In some families, however, salinity tolerance evolved early and has been broadly retained. For example, in Chenopodiaceae, adaptations such as succulence and other physiological mechanisms are derived from C_3_ lineages and have been conserved mainly in the evolved C_4_ salt-tolerant species (Kadereit et al., 2012). However, in other groups, with grasses as a prime example, there have been many independent origins of salinity tolerance, most of which are recent and result in only one or a few salinity-tolerant species each (Bennett et al., 2013; Moray et al., 2015). In most orders that contain halophytes, these comprise 1% or less of lineages, indicating a secondary evolution of the derived trait (Flowers et al., 2010). Thus, there is now general agreement that the most parsimonious scenario is that halophytes more commonly evolve independently in taxonomically diverse lineages (Bromham, 2015). For instance, the distribution of salt glands in over 50 plant species in several different families indicates that this innovation evolves repeatedly in species adapted to saline environments, not only to avoid Na+ and/or Cl− toxicity but also to regulate Ca2+ concentrations in the aerial tissues (Dassanayake and Larkin, 2017; Caperta et al., 2020). This raises a question: what underlies such convergence? There are good examples of what precedes it. For example, preadaptation to high salinity can be seen across the plant kingdom, with the required physiological or anatomical changes building rapidly on precursor traits acquired earlier (Moray et al., 2015). For example, grasses with C_4_ photosynthesis have a greater rate to gain and lose tolerance (Bennett et al., 2013), possibly because C_4_ increases water-use efficiency, limiting water stress and reducing ion uptake (Bromham and Bennett, 2014). Morphological specializations such as vivipary and aerial roots have also been seen as facilitating adaptation to harsh coastal environments in mangrove species (Shi et al., 2005). Therefore, it is important to not underestimate these latent traits that do not fit into the classical physiological mechanisms of salinity tolerance, because they can facilitate novel adaptations in plants evolving in saline environments.

The fact that salinity tolerance does tend to occur recently at the “tips” of phylogenies, rather than the bases, suggests some inherent cost, which may lead to reversion or eventual extinction (Bromham et al., 2020). This may also be linked to biogeography: although, in some saline regions, such as along coasts, salinity can persist for long periods; in others, salinity can vary over small spatial scales or shift at the population level seasonally (e.g., Busoms et al., 2018). If lineages are rapidly responding to fluctuating salinity (with high transition rates), this could partly explain why we infer mostly shallow gains of salinity tolerance that give rise to only one or a few extant halophytes (Bromham, 2015). Another explanation for why there are so many small clades of halophytes is that salinity tolerance may be costly (Munns et al., 2020) and thus difficult to maintain. For example, high phenotypic plasticity or capability could enable some lineages to transition into harsh novel habitats over evolutionarily short timescales (Edwards and Donoghue, 2013). However, maintaining salinity tolerance requires plants to produce osmolytes or investment in reactive oxygen species (ROS) scavenging and antioxidant production. Key enzymes in the detoxification of ROS are encoded by the RBOH genes. A recent review traced the evolution of salinity tolerance through changes in RBOH genes and showed a reduction in the number of isoforms to correlate with increases in salinity tolerance. Additionally, it showed that, rather than forge new proteins, salt-tolerant plants modified RBOH protein phosphorylation sites, which allows for improved activation of RBOH proteins (Liu et al., 2020b). This impressive efficiency contrasts with the general view that high physiological costs lead to increased extinction rates in halophytes, or high reversal rates of lineages that invest less in tolerance mechanisms have a strong competitive advantage. Such a view has been put forward to help explain why individuals from the same species adapted to coastal conditions perform more poorly in inland sites where conditions are usually more favorable to the species as a whole (e.g., Nagy and Rice, 1997).

## Evolutionary dynamics of adaptive salinity tolerance

Ecological specialization occurs primarily through local adaptation (VanWallendael et al., 2019), a process often required for successful establishment of populations in challenging new habitats. In this scenario, reproductive assurance (the ability to reproduce in small and/or isolated populations), and some prevention of gene flow from less fit relatives, are crucial. In an early work, Lowry and Willis showed that chromosomal inversions in *Mimulus* species contribute to reproductive isolation barriers between coastal and inland ecotypes of this species (Lowry and Willis, 2010). For the newly adapted population, a reproductive assurance can be gained by a transition to selfing during this time (Wright et al., 2013). However, outcrossers, especially obligate outcrossers, have high genetic variability, which, of course, facilitates adaptive evolution. Other phenological changes, particularly a shift in flowering time, also lead to reproductive isolation (McNeilly and Antonovics, 1968), boosting the likelihood that young adapted lineages may avoid influx of maladaptive genotypes from neighbors.

Halophyte species have evolved a range of adaptations to tolerate high concentrations of salts and colonize harsh environments (see Flowers and Colmer, 2015 for an excellent discussion). Thus, they can be a powerful genetic resource for biosaline agriculture. However, a lack of genomic information and low genetic similarity to major crops have compelled a focus on generic physiological mechanisms or particular gene variants that might be introduced in salt-sensitive species (Shabala, 2013; Abobatta, 2020). Despite a strong focus of modern research yielding advances on our understanding of adaptive mechanisms of halophytes (reviewed recently in Rahman et al., 2021), the molecular mechanisms of whole-plant adaptive responses to salinity are still unclear. A reason for this is that salinity tolerance in halophyte species is by definition constitutive to the entire species; thus, intraspecies variation is scant in halophytes, hindering, e.g., genome-wide association studies in discovering novel allelic candidates. That is why choosing non-halophyte species with contrasting within-species phenotypes in salinity tolerance is a particularly good approach for uncovering the mechanisms of ecologically adaptive salinity tolerance.

To date, local adaptation to high salinity has been typically associated with oligogenic architectures. In contrast to polygenic changes, which are defined by consisting of many genes with small effects, oligogenic indicates the involvement of few major-effect loci, with single alleles explaining up to 10% of the observed variation (Bell, 2009). For salinity tolerance, this often involves mutations of ion transporters and pumps (Volkov, 2015), either in their coding regions or mutations with effects on expression. It is thought that the type of genetic architecture (e.g., either oligogenic or polygenic) may be dependent on the type of environment and therefore the type of selection in a particular context (Whiteman, 2022). Accordingly, it is important to note that, despite our ability to explain large parts of this adaptive variation, in the cases where we have been able to find a major-effect locus underlying adaptation to high salinity, such as the *HIGH AFFINITY POTASSIUM TRANSPORTER* (*HKT1*) in *Arabidopsis thaliana* (An et al., 2017), the majority of the variation is still left unexplained and is likely due to the effects of many other genes.

Single-locus control of complex traits that do not obey a simple Mendelian inheritance pattern is uncommon, but blocks of linked genes, such as those associated with some types of structural genomic variation (SV; genomic variants >50 bp), are rapidly emerging as important in species subjected to environmental pressures (Zhang et al., 2021). For example, haplotype blocks associated with seed size, flowering time, and soil fertility in dune-adapted sunflower species were found to be highly divergent and associated with structural variants (Todesco et al., 2020). Also, natural variation (InDel) in the promoter of Gs*ERD15B* found in wild soybean affects the expression of this gene and others related to salinity tolerance mechanisms (Jin et al., 2021). Linkage among such variants may then be advantageous in loci under positive selection because it can allow the rapid, joint recruitment of multiple genes. However, under directional selection, local adaptation may also be based on successive recruitment of alleles at different loci, a process made possible by reduced gene flow (Llaurens et al., 2017). We do not yet have a good concept for how salt stress generally acts on recruitment of new “tolerance loci,” and further research should explore these concepts to shed more light here.

Contrary to traits under selection, where new adaptive combinations may rapidly replace ancestral ones, in traits under balancing selection, several alternative combinations may be maintained at relatively high frequencies, providing ample opportunity for recombination to adjust phenotypes by generating diverse combinations of polymorphisms (Delph and Kelly, 2014). Here, HKT1 also provides a clear example where we can see balancing selection in the context of adaptive evolution to increased salinity (Busoms et al., 2018).

It is now clear that even the frequency of *de novo* mutation varies considerably across the genome (Lynch et al., 2016; Monroe et al., 2022), with mutation bias holding broad consequences for our consideration of the mechanisms of evolutionary change. In fact, this mutation bias can interact with salinity specifically: in controlled conditions, Jiang et al. (2014) found that, even in short-term mutation accumulation experiments of less than a dozen generations, *A. thaliana* subjected to salinity stress accumulated twice as many mutations, and that these mutations actually exhibit a distinctive spectrum. In particular, they accumulated around 45% more differentially methylated cytosine positions at CG sites (CG-DMPs) than controls, and stress-associated CG-DMPs arose more frequently in genic rather than in non-genic regions of the genome. Further, Lu et al. (2021) concluded that heat stress over multiple generations accelerated mutation accumulation in intergenic regions, coding regions, and transposable elements, as well as non-synonymous mutations in functional genes. These results suggest that commonly encountered environmental stresses can accelerate the accumulation of mutations and change the profiles of novel variants.

Importantly, work to date has focused on SNPs rather than SV; even so, some of the clearest cases of adaptive evolution to edaphic stressors are SV, such as HMA4, HKT1, and MOT1 (Hanikenne et al., 2013; Busoms et al., 2018, 2021). For example, in the case of HMA4, a gene triplication set the stage for positive selection at the promoter region of this gene that results in elevated levels of gene product, improving heavy metal tolerance (Hanikenne et al., 2013). Different structural variants of HKT1 were associated with habitats close to the sea, and thus salt (Busoms et al., 2018), and deletions and duplications around the MOT1 gene have been associated with biomass and fitness changes under salinity stress (Busoms et al., 2021). Accordingly, we predict that the currently estimated impact of SV is greatly underestimated, and this will change once efficient population-level SV assessment is broadly applied. This time is not far off: pangenome approaches have recently made great advances with the improvements in both sequencing technologies (Campoy et al., 2020; Della Coletta et al., 2021; Meier et al., 2021) and approaches for the construction of graph-based multiple reference frameworks to incorporate SV diversity into references themselves (Garrison et al., 2018; Sirén et al., 2021). Such approaches use multiple, high-quality reference assemblies in a single graph-based representation, allowing efficient representation of SV across many genomes. To these genome graphs, alignment of large panels of sequenced populations provide information about allele frequencies of SV in populations (Bayer et al., 2020). Approaches such as these have already been useful to study general evolutionary processes (Qin et al., 2021) and are illuminating the hitherto dark zone of SV in many plant systems (Zhou et al., 2019, 2022a, and 2022b; Liu et al., 2020a; Alonge et al., 2020; Song et al., 2020; Cai et al., 2021; Hämälä et al., 2021).

## Benchmark studies defining mechanisms of salinity adaptation

The matching of an organism’s genome to the environment optimizes fitness to local habitat. Such genomic adaptation is driven by selective pressures acting at discrete geographic locations over dynamic timescales, and it is governed by a set of rules that allow life to optimize exploitation of a highly heterogeneous world. Both Wallace and Darwin recognized this, with Wallace noting “nothing can be more abrupt than the change often due to diversity of soil, a sharp line dividing a pine or heather-clad moor from calcareous hills” (Brady et al., 2005). Although over a century has passed since these observations, a systematic understanding of the molecular mechanistic basis of genomic reconstruction across species still eludes us.

In part, this is due to pervasive confounding effects of demography on studies of adaptive variation. Substantial recent progress has been made by high-density sampling at a local scale, largely controlling for the effects of demography. This retains contrasting phenotypes to as small a geographical region (and thus genetic dispersion) as possible (for a description of what makes a “benchmark study,” see box 2). For example, in a study of fine-scale local adaptation of *A. thaliana* plants in the Iberian Peninsula, a clear signal of salinity adaptation emerged over a distance of only 30 km, as shown in reciprocal transplant experiments (Busoms et al., 2015). In this study, both reciprocal transplant experiments done in several years (as well as common garden experiments) confirmed local adaptation to coastal and later also to inland soils (Terés et al., 2019). However, it is worth noting that such an experiment does not constitute formal proof for adaptation to high salinity, as inland and coastal soils vary for additional physiochemical properties. Thus, to isolate the specific impact of Na^+^, salinity stress experiments were performed in both soil and hydroponics. These revealed that plants from coastal habitats have increased tolerance specifically to elevated NaCl, establishing that elevated salinity in coastal soils is a key selective agent driving local adaptation (Busoms et al., 2015).

Box 1 **Salinity adaptation-relevant evolutionary terminology.**

**Figure 1:**
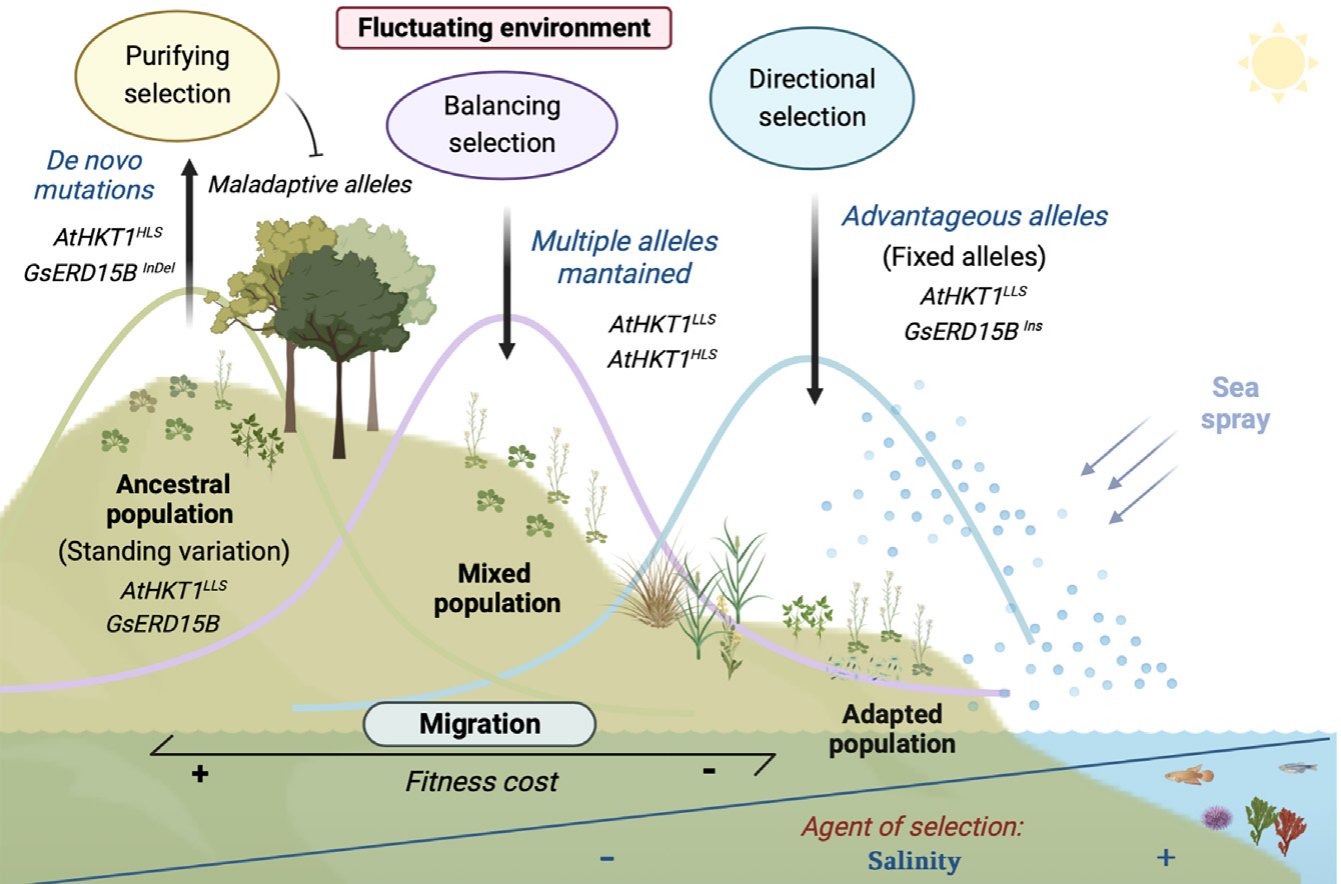
Schematic of mechanisms of adaptive salt tolerance. Consider the pictured landscape. Seawater provides a source of sodium ions; wind carries sea spray inland, creating a gradient of soil salinity. Ancestral populations of wild plant species originated inland. In this population, we observe standing variation, which is affected by *de novo* mutation and purifying selection, removing any alleles that come with a fitness disadvantage, or, in other words, that are maladaptive (e.g., *AtHKT1*^*HLS*^). Plant populations then by chance migrate to the seaside, possibly due to seeds being carried by humans or other animals. This derived population will represent a subset of the standing variation observed in the ancestral population. If it carries some of the rarer alleles, which are under purifying selection further inland, due to the high cost associated with them, these alleles could now be under positive selection if they are adaptive in the new location. These alleles would become fixed in this new habitat (e.g., *GsERD15B*^*Ins*^). Under this scenario the effective population size decreases, the phenotype becomes much more constant, and plasticity is reduced. Other realistic scenarios include migrants harboring these alleles at a much higher frequency representing stepping stones in that direction. In these migrants, balancing selection maintains a relatively high frequency of an allele. This could reflect the allele being required at certain times in the year or in certain challenging but regular events (e.g., mixed population of *AtHKT1*^*HLS*^*and AtHKT1*^*LLS*^).

Genetic diversity depends in part on “*de novo mutations”* entering a population and their effect on fitness. Most novel mutations are thought to be either deleterious or neutral, but occasionally they can be beneficial (e.g., Jin et al., 2021). See Figure 1 for these terms in genographic and adaptive context.

“Directional selection” can cause advantageous alleles to become more frequent in a population, driven by a selective advantage. Directional selection often reduces the diversity of alleles around a causative locus, and therefore, at least briefly, the genetic variation in a population, in the form of bottlenecks. But this reduction in local genomic diversity is of course beneficial when it leads to “local adaptation.” Here, for example, when salinity acts as an agent of directional selection favoring alleles that allow plant survival in coastal habitats (e.g., Busoms et al., 2015).

“Purifying selection” is a prevalent form of natural selection that constantly removes deleterious mutations. However, purifying selection is weak enough for some mutations to be able to establish themselves in the population if purifying selection is of the same order or lower than genetic drift. Where purifying selection is weak, “standing variation” is increased, providing a substrate upon which selection may act (e.g., Wang et al., 2021).

“Genetic drift” is the change in allele frequencies that occurs mainly in smaller (or inbred) populations due to the random sampling of alleles. Genetic drift can be distinguished from selection because the entire genome is generally affected, not only a single locus. It is worth noting in respect to salinity adaptation that it is very likely that isolated populations suffer genetic drift, which counters the maintenance of adaptation to salinity, with little alternative but to migrate inland or go extinct (e.g., Prinz et al., 2013).

“Migration” is a counteracting force to genetic drift. By mixing alleles among populations, migration distributes and homogenizes genetic variation across species ranges, countering strong directional selection and bottlenecks. Migration can also contribute to “negative frequency-dependent selection,” favoring rare immigrants over locally adapted plants (e.g., Posavi et al., 2014).

“Balancing selection” occurs when multiple alleles are maintained in a population, which can result in their preservation over long periods. Such selection occurs in intermediate-salinity sites or fluctuating environments, and it allows two or more allelic groups to be maintained in a population, in many cases reproductively isolated, at the same site (e.g., Busoms et al., 2018).

Box 2 **Benchmark Approaches to define local adaptation.**

Where tested, the sum output of ecological and genetic factors, “local adaptation,” has been broadly observed. For example, a meta-analysis of 32 species showed that local plants outperform foreign plants in 71% of cases (Leimu and Fischer, 2008). Clear divergent selection was observed in a more stringent comparison between both environments in 45% of cases. This effect is best observed in large populations, suggesting that smaller populations lack sufficient genetic diversity for rapid adaptation. However, what is a sufficient definition for local adaptation? Only where local plants outperform foreign plants in both habitats under investigation can we specify local adaptation of both genotypes. If one plant outperforms another in both habitats, however, true local adaptation cannot be inferred. This is further supported by Nuismer and Gandon (2008), who show by modeling that only reciprocal transplant experiments are capable of measuring local adaptation. They attribute this to the properties of common garden experiments to measure only spatial covariance between genotype frequencies. Reciprocal transplant experiments, on the other hand, incorporate spatial variability in the ecological environment as a further term.

Local adaptation to salinity stress is often studied in reciprocal transplant experiments in coastal and inland habitats. Growth and fitness are then compared to assess relative performance of all plants. Growth is thereby an indirect measure. Ecologically relevant fitness captures the ability of individuals to transmit their genotypes to following generations, by estimating the number of fertile progenies an individual can produce in prevailing conditions. Common garden experiments are often used, frequently in addition to reciprocal transplants, to infer the presence of locally adaptive evolutionary change. In a common garden experiment, plants of differing origins are grown at a single site. These can be in controlled environments, such as greenhouses or growth chambers, or in the field. Unlike in a reciprocal transplant experiment, the effect of the environmental variation on fitness is not assessed, unless multiple gardens are used. In each case, the impact of genetic variation on phenotype can then be estimated. An overview of published plant reciprocal transplant and common garden experiments is given in Table 1.

**Table 1:** An overview of published plant reciprocal transplant and common garden experiments.

Species	Type of experiment	Years	Environment	Medium	Factor	Evidence for local adaptation	Candidate loci	Author
*A. thaliana*	reciprocal transplant	2 years	in field	*in situ* soil	shade	no	no	Callahan and Pigliucci, 2002
	common garden	1 year	controlled environment	potting mix				
*A. thaliana*	reciprocal transplant	1 year	in field	*in situ* soil	dune vs. inland	local over foreign	no	Arany et al., 2009
*A. thaliana*	common garden	1 year	in field	*in situ* soil	latitude, oceanic vs. continental	alleles with lower fitness had greater climate specialization: specialized alleles for special climate: local adaptation	LAC1, AT1G18130, CHR8, AT2G18780, PHYB, delta-TIP, NDF4, TRZ4, AT3G16270, SAG21, AT4G02370, PARP1	Fournier-Level et al., 2011
*A. thaliana*	reciprocal transplant	3 years	in field	*in situ* soil	north Sweden vs. south Italy	local over foreign	15 QTLs	Ågren and Schemske, 2012
*A. thaliana*	reciprocal transplant	2 years	in field	*in situ* soil	soil salinity	local over foreign	*HKT1*	Busoms et al. (2015)
*A. thaliana*	response to Na treatment	2 years	controlled environment	*ex situ* soil	NaCl	not studied	15 genes, AT4g08850, MUSTANG1, AT1G25370	Julkowska et al., 2016
*A. thaliana*	reciprocal transplant	2 years	in field	*in situ* soil	soil salinity	local over foreign	*HKT1*	Busoms et al. (2018)
*A. thaliana*	reciprocal transplant	2 years	in field	*in situ* soil	Coastal vs. inland	local over foreign	*MOT1*	Busoms et al. (2021)
	reciprocal transplant	2 years	controlled environment	*ex situ* soil	NaCl			
*A. thaliana*	common garden	several	in field	*in situ* soil	latitude, oceanic vs. continental	not studied	flowering time control for FRI, GIS5, PKT4, and RDO5	Fournier-Level et al., 2022
*Avicennia schaueriana*	common garden	1 year	controlled environment	sand: *in situ* soil	latitude, water deficit, and solar radiation	local over foreign	loci associated with photosynthesis, anthocyanin accumulation, responses to osmotic stress and hypoxia	Cruz et al., 2019
*Borrichia frutescens*	common garden	1 year	controlled environment	sterilized sand: organic medium	NaCl	no	no	Richards et al., 2010
*Camissoniopsis cheiranthifolia*	reciprocal transplant	1 year	in field	*in situ* soil	latitude, coastal	no	no	Samis et al., 2016.
*Gilia capitata*	reciprocal transplant	3 years	in field	*in situ* soil	coastal vs. inland	local over foreign	no	Nagy and Rice (1997)
*H. bonariensis*	reciprocal transplant	1 year	in field	*in situ* soil	height and low dune with salt gradient	local over foreign if local vegetation was maintained	no	Knight and Miller (2004)
*M. truncatula*	reciprocal transplant	1 year	in field	*in situ* soil	soil salinity	local over foreign	*CIPK21* ortholog; trehalose-6-phosphate phosphatase, regulators of ABA and JA, CPK ortholog	Friesen et al. (2014)
	common garden	1 year	controlled environment	*in situ* soil				
	common garden	1 year	controlled environment	sterile sand				
*Mimulus guttatus*	reciprocal transplant	1 year, four locations	in field	*in situ* soil	coastal vs. inland	local over foreign	no	Lowry et al. (2008)
	common garden	1 year	controlled environment	potting mix	NaCl			
*Mimulus guttatus*	manipulative reciprocal transplant	1 year	in field	*in situ* soil	coastal vs. inland	costal ecotype outperformed inland ecotype	no	Popovic and Lowry (2020)
*Oryza coarctata*	Response to Na treatment	2 years	controlled environment	unknown soil with saline river water	NaCl	not studied	no	Bal and Dutt, 1986
*P. australis*, *S. alterniflora*	common garden	1 year	controlled environment	mixture of peat and *in situ* soil	NaCl	foreign over local in a changing habitat	no	Vasquez et al. (2006)
*Porteresia coarctata*	Response to Na treatment	1 year	controlled environment	potting mix	NaCl	not studied	no	Flowers et al., 1990
*T. purpurea*	common garden	1 year	controlled environment	sterile sand: *in situ* soil	sea salt spray	no	no	Cheplick and White (2002)
*Zea mays*	Common garden	1 year	controlled environment	potting mix	NaCl	not studied	*HKT1*, *HAK4*	Zhang et al. (2018 and 2019)
32 plant species	reciprocal transplant	varying	Varying	varying	various	local over foreign	no	Leimu and Fischer (2008)

In coastal areas, salinity challenges come in two major physical modalities: aboveground due to salt spray and belowground due to soil salinity (Du and Hesp, 2020). Popovic and Lowry (2020) implemented a manipulative reciprocal transplant of *Mimulus guttatus* in coastal and inland sites excluding aboveground stressors. They found that inland plants cultivated in the coast but protected with enclosures exhibited the same fitness than in inland sites, proving the importance of salt spray effects. This suggest that, in this system, most of the salt enters the aerial organs of plants due to long-term exposure to salt spray (Lowry et al., 2009). Once the salt has entered the leaf tissue, most of it is translocated to the tips of leaves, accumulated or compartmentalized there, loaded to the phloem, or secreted using the same mechanisms employed to remove an excess of salt translocated from the roots (Tester and Davenport, 2003). Tolerance to salt spray increases with the growth of vegetation because well-developed cuticles prevent salt penetration. The exception is that reproductive organs are usually much more sensitive to salt spray than plant leaves (Griffiths, 2006) and therefore escape strategies can be essential. Additionally, it is important to note that various coastal species have evolved particular traits to avoid salt stray injury (see Maun, 2009), including morphological and hormone signaling changes affecting the growth habit. For example, the coastal short ecotype of *Setaria viridis* exhibits higher salt spray tolerance than the coastal tall ecotypes because the compact stature offers major protection to the strong winds from the open sea (Itoh, 2021). Relating adaptive changes in stature to a basis in hormone regulation, Wilkinson et al. (2019) showed that differences in the auxin pathway contributed to the repeated evolution of erect and prostrate forms of *Senecio lautus* along the Australian coast.

We speculate that whole-plant changes in structure, habit, and physiology require the modification of multiple loci; what about single major-effect natural changes? Worldwide, natural alleles of the HKT1 gene are the single greatest component explaining variation in leaf Na^+^ accumulation in *A. thaliana* (Baxter et al., 2010). HKT1 is a Na^+^ transporter that functions to recycle Na^+^ out of the xylem and restricts Na^+^ transport to the shoot (Horie et al., 2009). Indeed, an *HKT1;1* variant that is only weakly expressed in roots and associated with elevated leaf Na^+^ is enriched in coastal regions, including from the coastal region in the Iberian Peninsula (Baxter et al., 2010). The coastal allele of *HKT1;1* was shown to have enhanced shoot expression, which protects the inflorescence from excessive Na^+^ accumulation (An et al., 2017), further suggesting mechanistic roles in coastal adaptation. However, the *HKT1;1* story is not so simple as a binary phenotype. Extending this thread, a quantitative response was established, with the coastal allele of *HKT1;1* being in fact maladaptive to the highest soil Na^+^ concentrations found directly along the coastline. Instead, this adaptive coastal allele occurs only in plants ∼500–1500 m from the sea, where soil salinity is intermediate and strongly influenced over short timescales by rainfall levels. Further, at these locations, this allele is under dynamic year-to-year fluctuating selection due to oscillating soil salinity driven by annual variation in rainfall (Busoms et al., 2018).

Moving beyond *A. thaliana*, *HKT1* has also been shown to explain interspecific variation in Na^+^ acquisition in crops, and to alter yield under Na^+^ stress (Kotula et al., 2020). In particular, studies of Na^+^ content and tolerance in barley (Hazzouri et al., 2018; van Bezouw et al., 2019), rice (Zhang et al., 2018, 2019), and wheat (Byrt et al., 2007) point to *HKT1* as a broadly flexible gene modulating salinity-related phenotypes across both monocots and dicots. However, it has not yet been studied how extensively variation in this locus serves a natural, adaptive evolutionary function, as it does in *A. thaliana*, because all the crops noted above were strongly subject to artificial selection.

Fascinatingly, a clear parallel to the *HKT1;1* story emerged in the same fine-scale “natural laboratory in the Iberian Peninsula. However, in this case, the locus primarily controlled molybdenum accumulation, with additional pleiotropic effects on copper and sodium. There, naturally evolved variants of the molybdenum transporter *MOT1* were analogously associated with coastal adaptation (Busoms et al., 2021). In a worldwide sampling, natural variation at *MOT1* explains a high proportion of the global, species-wide variation in leaf molybdenum in *A. thaliana* (Forsberg et al., 2015). Also, strikingly similar to the case of *HKT1;1*, a natural deletion in the promoter of the *MOT1* transporter leads to low expression of the allele (Baxter et al., 2008), a weak allele of *MOT1*, which appears to mediate adaptation to coastal habitats (Busoms et al., 2021). Here also, the low-expressing allele was only found within <3 km of the coast, and reciprocal transplants demonstrated enhanced fitness specific to the coast. Mechanistically, the *MOT1* variant harboring this SV, a promoter deletion, appears to be part of a complex crosstalk between Mo, Cu, and Na^+^. This results in enhanced Cu uptake, and improved formation of Moco—an essential co-factor in ABA biosynthesis that promotes ABA production—helping reduce Na^+^ accumulation. It is worth noting, however, that both examples are not completely similar. The variant of the *HKT1;1* allele is likely using Na^+^ as a cheap osmoticum to enable plants to maintain water and ion transport at elevated levels of soil sodium (Munns and Tester, 2008). In contrast, the variation observed in *MOT1* leads to an indirect adaptation to soil salinity through ABA signaling, promoting Na^+^ efflux and water uptake as observed in other species (e.g., Kong et al., 2016).

Further molecular insight into mechanisms of adaptive salinity tolerance has often come from genomic association studies. A particularly well-studied case concerns the distribution of *Mimulus guttatus* along the west coast of the USA (Lowry et al., 2008). In a reciprocal transplant experiment, the authors compared coastal and inland individuals of *M. guttatus*, which have a strongly differentiated population structure. They showed that local plants consistently outperformed foreign plants in survival, as well as the fitness proxies number of flowers and growth. They related at least part of this effect to sea spray by showing high damage in inland plants, relative to more tolerant coastal plants. A follow-up study described the genome-wide differentiation between the coastal and inland plants (Gould et al., 2017), highlighting differentiation for two large SV: chromosomal inversions. In these regions shielded from recombination the frequency of non-synonymous changes was elevated, and the authors suggested plausible candidate genes that may underlie the observed adaptive differences. Although this has not been shown in follow-up studies as none of the candidate alleles has been functionally confirmed, it implies that the SV in this case may underpin salinity tolerance. As this study focused on speciation, the authors do not draw any conclusion about the mechanism of salinity tolerance per se, but they do point to salt and drought stress response genes, gibberellic acid signaling, and developmental genes as possible candidates as mediating local adaptation.

In the context of very-high-salt endemics, traits related to higher tolerance were the focus of a study of halophytes growing along a natural gradient of salinity (Howard, 2010; Rouger and Jump, 2015). There, *Haloxylon aphyllum* populations showed varying morphological and physiological adaptations in different genotypes, which all indicated salinity tolerance. For example, the authors detected high levels of K^+^ under all levels of salinity stress in plants that were able to maintain a steady growth under increasing salinity. They also showed evidence that higher proline levels were beneficial at the highest salinities. These different adaptations were interpreted to underlie contrasting mechanisms of salt tolerance (Shuyskaya et al., 2014). Here, interestingly similar to the *HKT1* scenario, the authors found the greatest genetic diversity at intermediate Na^+^ levels (Shuyskaya et al., 2012), indicating the ability to select genes useful for performance on either higher or lower levels of salinity.

Candidate genes underlying adaptation to coastal environments have been identified in various studies, but, interestingly, high salinity is not always the factor best linked to the adaptations. Other traits are sometimes better correlated with occurrence in high-saline areas. Studies on these could illuminate different, important selection pressures related to adaptation to high-salt areas, where the mechanism of apparent salinity tolerance is more related to mitigating deleterious effects of the environment in general. For example, coastal areas are often unsurprisingly less arid habitats than inland, and coastal *M. guttatus* have larger leaves, more branches, and greater overall size, and flower later (Lowry et al., 2008). This syndrome is broadly related to marine habitats, as it corresponds to a higher photosynthetic rate, which comes at the cost of high water loss, which is of course detrimental when water is less available (Stebbins, 1952; Hayford et al., 2022). Indeed, three of the adaptive candidate genes detected by Gould et al. (2017), ent-Kaurene oxidase (*KO*), AGAMOUS-like 8 (*AGL8*), and auxin response factor 8 (*ARF8*), co-localize with quantitative trait loci (QTLs) for flowering and developmental traits (Hall et al., 2006). *KO* is involved in gibberellic acid (GA) biosynthesis (Helliwell et al., 1999), *AGL8* is expressed in shoot meristems and is, together with other factors, responsible for the initiation of flowering (Hempel et al., 1997), and *ARF8* promotes jasmonic acid (JA) production and is, together with *ARF6*, essential for flower maturation (Nagpal et al., 2005). Further candidates also relate to GA metabolism, flowering and auxin, as well as brassinosteroid signaling and ABA synthesis (Gould et al., 2017). ABA, as previously mentioned, is a phytohormone to signal stress and, in the case of salt, it promotes Na^+^ efflux and water uptake (Kong et al., 2016). The signaling hormone has been shown to act through late embryogenesis abundant (LEA) proteins, which are upregulated by ABA and whose high abundance leads to salinity tolerance (Dalal et al., 2009). Alternatively, in an ABA-independent mechanism, dehydration-responsive element binding (DREB) genes are known to regulate many downstream targets during salt stress (Yan et al., 2014), but no evolutionary signal for selection has yet been found for DREBs. Neither has such a role been found for other signaling compounds such as nitric oxide (NO) or small molecules such as polyamines. These have been shown to also protect against salinity. Polyamines are required for Ca signaling, which is important in reducing salt toxicity symptoms (Yamaguchi et al., 2006). Interacting with polyamines is NO, which is required for post-translational modifications on proteins and subsequent changes in enzymatic activities, and gene expression changes, which have been correlated with salt stress responses (Napieraj et al., 2020). The phytohormone GA, with its impact on plant morphological parameters, is able to promote growth under Na stress (Wen et al., 2010).

Genes such as *ARF8* and genes related to GA signaling are all likely connected to other phenotypic adaptations to coastal areas, such as early flowering (*ARF8* and *KO*) and changes in morphology (GA). However, evidence for divergence in the genome of coastal and inland *M. guttatus* was also detected for genes involved in ion homeostasis. Such genes, or their promotors, were in the top 1% of the most differentiated genes between coastal and inland plants of *M. guttatus*. Among them were *SALT OVERLY SENSITIVE 1* (*SOS1*) and *SOS3*, two members of the well-described SOS pathway (Quintero et al., 2002) for Na^+^ tolerance. Additionally, divergence for *HKT1* alleles was also detected (Gould et al., 2017).

Further convincing evidence of population-level, within-species salinity adaptation has been seen in *Medicago truncatula* sampled across a salinity gradient in Tunisia (Friesen et al., 2014). Populations originating from saline sites proved to be locally adapted, displaying higher biomass in high-salinity common gardens as well as in reciprocal transplant experiments. The authors showed that traits such as increased leaf water content, and early germination and flowering, are favored in populations from saline soils. Integrating genome scans with ecological experiments and selection analysis, 16 genomic regions and 198 candidate genes were linked to the soil of origin, and therefore potentially underpin local adaptation to high saline soil. Among these candidates there are ABA and JA regulators, as well as a gene involved in trehalose metabolism that could function in osmotic protection. Importantly, the researchers also discovered a *CIPK* gene, orthologous to *CIPK21*, as well as Ca^2+^ signaling candidates such as calcium protein kinases. This supports the interesting idea that Ca^2+^ signaling may be adaptively tuned. Given the central role of Ca^2+^ in stress signaling, the idea that adaptive modulation of Ca^2+^ transport may act as a more global molecular rheostat in stress signaling was speculated by Arnold et al. (2016), who observed convergence on multi-hazard—and severely Ca^2+^-challenged environments—of serpentine sites. This idea was later supported by the discovery of remarkably specific, convergent *de novo* substitutions in the selectivity gate of the central Ca transporter and stress signaling hub TWO PORE CHANNEL 1 only on serpentine sites (Konečná et al., 2020), despite strict conservation at that residue across plant diversity (and indeed homologs in other kingdoms).

The above studies provide generally clear evidence for the mechanisms (both evolutionary and molecular) underlying adaptation to salinity and related ionomic challenges. However, most often information on the mechanisms for underlying adaptive traits is still missing, especially in less established model systems. This is a real shortcoming, since other wild species than e.g., *A. thaliana* harbor the greatest potential for understanding salinity adaptation. This can be seen in a variety of ecological studies. For example, *Hydrocotyle bonariensis* showed local adaptation as defined by Leimu and Fischer (2008) between high dune areas, further away from the water edge and with generally dryer conditions with less vegetation, and low dune areas, which are often flooded (Knight and Miller, 2004). The species had been shown to occur in heterogeneous environments, including steep soil saline gradients from 0.5% to 16% (Evans and Whitney, 1992). This interesting work did not fully dissect the basis of local adaptation, but, given the salinity gradient naturally present within the environment, it is likely that each local population is adapted to the soil salinity level. The same is true for the relatively salt-tolerant *Triplasis purpurea*, which provides an interesting counterexample. There, different populations were subjected to varying degrees of sea salt spray but did not differ in traits such as tiller number and biomass (Cheplick and White, 2002). In contrast, the authors found a phylogenetic family effect for most traits they measured, which indicated a genetic relatedness. Consequently, instead of showing selection at a particular locus, this indicates plants were only recently derived from a common ancestor. This means that plants are not yet adapted to high salinity but rather respond differently to salinity stress based on different allele combinations inherited by their parents. Such patterns are less likely due to local selection but rather demographic history, and they hold the opportunity for rapid adaptations.

As mentioned above, local adaptation is often required when migrants experience a new or challenging habitat. Invasive species have a knack for this and, consequently, we find clear examples of local adaptation to salinity among them. On the other hand, invasive species can exhibit generalist strategies, and/or plasticity. This then raises a question: what might be common evolutionary or molecular mechanisms to be shared by salt-tolerant invasive species? Such integrative, comparative studies are very rare, but one work compared two invasives, *Phragmites australis* (which is invading North American salt marshes that are normally the home of *Sporobolus alterniflora*), and *Sporobolus* spp. (derived from *S. alterniflora*, which is invasive in European marshes home to *P. australis*), in terms of salinity tolerance (Vasquez et al., 2006). In common garden experiments, *S. alterniflora* produced much more biomass at higher NaCl than *P. australis*. In contrast, at low NaCl, *P. australis* had more rhizomes than *S. alterniflora*, indicating potentially higher rates of vegetative reproduction in low saline environments. North America’s salt marshes are experiencing a reduction in their salinity, potentially favoring *P. australis* and allowing it to become invasive. Further examples of invasive salt-tolerant species include *Spartina alterniflora*, a perennial grass native to North America but invasive in south China. Here, *S. alterniflora* is disrupting mangrove ecosystems due to its high salinity tolerance, which is connected to increased production and signaling through hydrogen sulphate. This mitigates damage from ROS and helps to maintain Na^+^/K^+^ homeostasis (Li et al., 2020). Similar mechanisms were also part of the tolerance strategy of *Acacia longifolia*, an invasive species in Portuguese sand dunes, which copes better with Na^+^ stress through higher K^+^/Na^+^ ratio and higher ROS scavenging capacity (Morais et al., 2012). Many more examples exist (Rouifed et al., 2012; Gonzalez-Mateu et al., 2020); however, in most studies, mechanistic insights into the Na^+^ tolerance of invasives are still missing and no broad-scale comparisons have been performed.

Extreme salinity may even enable invasion, as is currently occurring in *Cochlearia danica*, a recently-formed allohexaploid. This species, an Atlantic coastal halophyte, is spreading exceptionally rapidly along major motorways across Europe, triggered by the widespread use of salt-based road de-icing since the 1970s. The mechanism of their extreme salt tolerance is unknown, and we do not yet know for certain if salt tolerance in this case means sodium tolerance specifically. However, it has been shown that *C. danica* seeds can germinate at very high sodium concentrations (Pegtel, 1999), allowing the rapid invasion of competitor-sparse habitats (Fekete et al., 2018).

## Thus-far-discovered mechanisms

Taking the work discussed above as a whole, the primary molecular mechanisms for salinity tolerance can be grouped into three non-exclusive categories—osmotic stress tolerance, ion exclusion, and tissue tolerance—and all have been excellently described, especially in mutant and crop studies (e.g., Munns and Tester, 2008; Almeida et al., 2017). Explicit discussion of evolutionary mechanisms is more difficult to find. Additionally, the genomic basis of these mechanisms has been mainly studied in model plants such as *A. thaliana*. Luckily, despite being considered a glycophyte, there are wild populations of *A. thaliana* with contrasting salinity-tolerance phenotypes. This fact has allowed the discovery of natural variants such as *HKT1* (Baxter et al., 2010) through genome-wide association analysis (GWA). In the past decade, GWA and QTL studies have enabled progress in the identification of major-effect genes controlling salt tolerance (Li, 2020; Wani et al., 2020). As a fascinating example of evolutionary convergence, rice *SKC1* (Ren et al., 2005), wheat *Kna1* (Munns et al., 2012), *Nax1* (Byrt et al., 2007) and *Nax2* (Huang et al., 2006), and maize *ZmNC1* (Zhang et al., 2018) salt-tolerant QTLs are all based on *HKT1* homolog-mediated mechanisms. Additionally, tomato *SlHAK20* (Wang et al., 2020) and maize *XmHAK4* (Zhang et al., 2019) are members of the HAK/KUP/KT Na^+^-selective ion transporters that promote shoot Na^+^ exclusion and confer salinity tolerance.

Early work on the SALT OVERLY SENSITIVE (SOS) pathway pioneered the molecular understanding of salinity tolerance in *A. thaliana* (Wu et al., 1996). The SOS pathway is broadly essential for salinity tolerance, and is conserved functionally across dicots and monocots. Strikingly, however, evidence for natural adaptive genetic variation in SOS genes is minimal. For example, large-scale GWA studies (GWASs) in *A. thaliana* (Baxter et al., 2010; Almira Casellas et al., 2023), rice (Lv et al., 2022), maize (Luo et al., 2019), and barley (Tu et al., 2021) have not detected putatively adaptive variation in SOS genes. This stands in contrast to HKT1, which exhibited repeated adaptive variation to natural salinity challenge (Rus et al., 2006; Baxter et al., 2010; Zhang et al., 2018, Busoms et al., 2018). While the degree of adaptive flexibility at HKT is much greater, we note that, in association with domestication, deleterious hypomorphic or loss-of-function SOS alleles have been observed; for example, during the domestication of tomato (Wang et al., 2021) and maize (Zhou et al., 2022a; 2022b). Interestingly, the well-characterized adaptive “weak allele” of HKT1 shares with these SOS alleles low expression, but, crucially in HKT1, this low expression has been associated with adaptive value against elevated salinity in nature, while in SOS this has not been observed.

GWASs have also been integrated with mutant analysis, expression networks, and other “omic” techniques to identify promising genes. For example, Tu et al. (2021) identified 39 salt-responding genes in barley, including the salt signaling genes *CYPs*, *LRR-KISS*, and *CML*, integrating GWA and RNA sequencing (RNA-seq) analysis. However, given limitations in power, all current approaches are biased toward discovering the largest effect loci, and thus relatively oligogenic architectures. This is, of course, a bane across studies of adaptation, but is slowly being overcome by novel approaches and increased power in, e.g., very-large-scale association studies. Such studies typically provide a much more locally refined picture of genetic variation and therefore enable more meaningful genotype environment or subpopulation correlations. They also increasingly include complementary data types and analysis, such as the prediction of tertiary protein structures, network analysis, or interactomes (e.g., Wu et al., 2021). Increasing application of such analyses to non-standard models will provide greater insight into a broader array of adaptive mechanisms.

## A rare, salient role for salt adaptation in polyploids?

The product of whole-genome duplication (WGD), polyploidy occurs prevalently across the plant kingdom (Cui et al., 2006; Wood et al., 2009; Alix et al., 2017) and leads to instant speciation. The immediate physiological effects of WGD, however, are notoriously idiosyncratic (Yant and Bomblies, 2015; Doyle and Coate, 2019; Bomblies, 2020). Most obviously, WGD instantly allows for doubled mutational targets, freeing up genetic material for novel innovations. However, given time, polyploids eventually revert back to diploidy. However, before that occurs, they typically accumulate mutations resulting in adaptive phenotypes, and subsequently often niche shifts/expansions, along with sometimes increased colonization potentials.

While we strongly underscore that every polyploidy event generates variable phenotypes, there appears to be a tendency for neopolyploids to exhibit some fairly common, ecologically relevant benefits (reviewed recently by Baduel et al., 2018; Bomblies, 2020). Increased salinity tolerance is perhaps the clearest among these. This was best shown in a panel of neo-tetraploid *A. thaliana* lines that were in all respects isogenic to their diploid counterparts, except for their laboratory-induced polyploidy. These early generations of autotetraploids exhibited higher seed production and survival under Na^+^ stress than their isogenic diploid sisters (Chao et al., 2013). This effect was concomitant with increased shoot K^+^ concentrations and an improved K^+^/Na^+^ ratio under Na^+^ stress. This effect was also shown in an established polyploid *A. thaliana* accession. Maintaining a balanced K^+^/Na^+^ ratio is important for Na^+^-stressed plants, because increased Na^+^ concentrations in root and shoot cells can displace other ions, most notably K^+^, from binding sites and inhibit cellular functions (Nitsos and Evans, 1969). We speculate that this immediately altered intracellular ionomic environment in young polyploids may act as an evolutionary spandrel, later serving as a trait that is then selected on when the nascent polyploid encounters novel environmental challenges. Supporting the argument that K^+^/Na^+^ homeostasis is important for polyploids to develop Na^+^ tolerance is that an improved K^+^/Na^+^ ratio also coincides with better Na^+^ tolerance in other systems, such as through mycorrhizal colonization of *Acacia nilotica* with *Glomus fasciculatum* (Giri et al., 2007). Interestingly, improved growth and decreased Na^+^ concentrations under salinity stress was also observed in neo-tetraploid rice (Tu et al., 2014; Wang et al., 2021), where the authors conclude that neo-tetraploids are better able to cope with the Na^+^ stress due to their increased vigor, and activated JAs controlled stress response. Further, the diploids *Brassica oleracea*, *Brassica campestris*, and *Brassica nigra* are less salinity tolerant than their amphidiploid (contains diploid sets of chromosomes from each parent) offspring *Brassica napus*, *Brassica carinata*, and *Brassica juncea* (Ashraf et al., 2001). The amphidiploids also accumulate higher concentrations of K^+^ under salinity stress. Moreover, tetraploid citrange also showed less leaf damage and defoliation after salinity treatment (Ruiz et al., 2016).

It is clear that ploidy increase can bring amplified salinity tolerance (Gerstein et al., 2006; Saleh et al., 2008). However, we do not yet understand the molecular mechanisms underpinning this. Genetic analysis of neo-tetraploid mutants indicates that increased shoot K^+^ concentrations are regulated through a gene network that is composed of hubs of endodermal and cell wall modification genes (Fischer et al., 2021). Population genomic analysis of polyploid, salt-tolerant *Cochlearia* populations revealed selective sweeps for the orthologs of *SOS1* and HKT1 in the autotetraploid relative to inland diploids (Bray et al., 2020). This Na^+^/H^+^ transporter is relevant for Na^+^ tolerance in *A. thaliana* (Quintero et al., 2002, 2011), and was also found to affect shoot potassium concentration in neo-tetraploid *A. thaliana* (Fischer et al., 2022). Bray et al. (2020) also elaborated that very similar processes (relevant to salinity: ion homeostasis), but not orthologous genes were under selection after WGD in *Arabidopsis arenosa* (Yant et al., 2013) and *Cardamine amara* (Bohutínská et al., 2021). These examples point to common, shared mechanisms—with ion homeostasis prominent among them—underpinning adaptation to the transformed intracellular WGD state. Indeed, tetraploid *A. arenosa* populations have been found on highly diverse soils, including mines and serpentines, if not explicitly saline environments. That said, dedicated studies failed to detect niche differentiation between diploid and tetraploid *A. arenosa*, although one showed niche expansion for the tetraploids (Molina-Henao and Hopkins, 2019; Morgan et al., 2020). Common garden experiments utilizing diverse cytotypes of many populations to capture variation, and natural soil with contrasting elemental profiles, will establish the impact of genotype and cytotypes on growth and the plant ionome. Reciprocal transplant experiments between sites with contrasting soil physiochemical properties together with cytology and genomic techniques will allow us to assess adaptive responses and study the molecular mechanisms behind the improved salinity tolerance of polyploids.

## A little help from friends

Complex interactions that evolved between plants and associated microbiomes are now well recognized as key determinants of plant health (Berendsen et al., 2012). The microbiome works with plants in obtaining nutrients, protecting against infections, and enduring stresses (Box 3). An array of recent studies highlight the importance of microbial communication with the plant, proposing mechanisms based on plant-microbe associations that accentuate plant defense (Petrić et al., 2022). Location, soil properties, and plant genotype have a significant effect on microbial communities (see Morales Moreira et al., 2021). Different microbial compartments (bulk soil, rhizosphere, and rhizoplane) also harbor contrasting microbial compositions due to the distance to the host root (e.g., Edwards et al., 2015). The soil microbiome is directly affected by environmental fluctuations, while rhizosphere microbiomes are influenced also indirectly by host responses (Trivedi et al., 2022). Endophytes are likely less affected by environmental fluctuation, as they occupy relatively more stable internal plant tissue environments, and they are typically more host specific.

Box 3 **Microbiome reciprocal transplants.**

**Figure 2:**
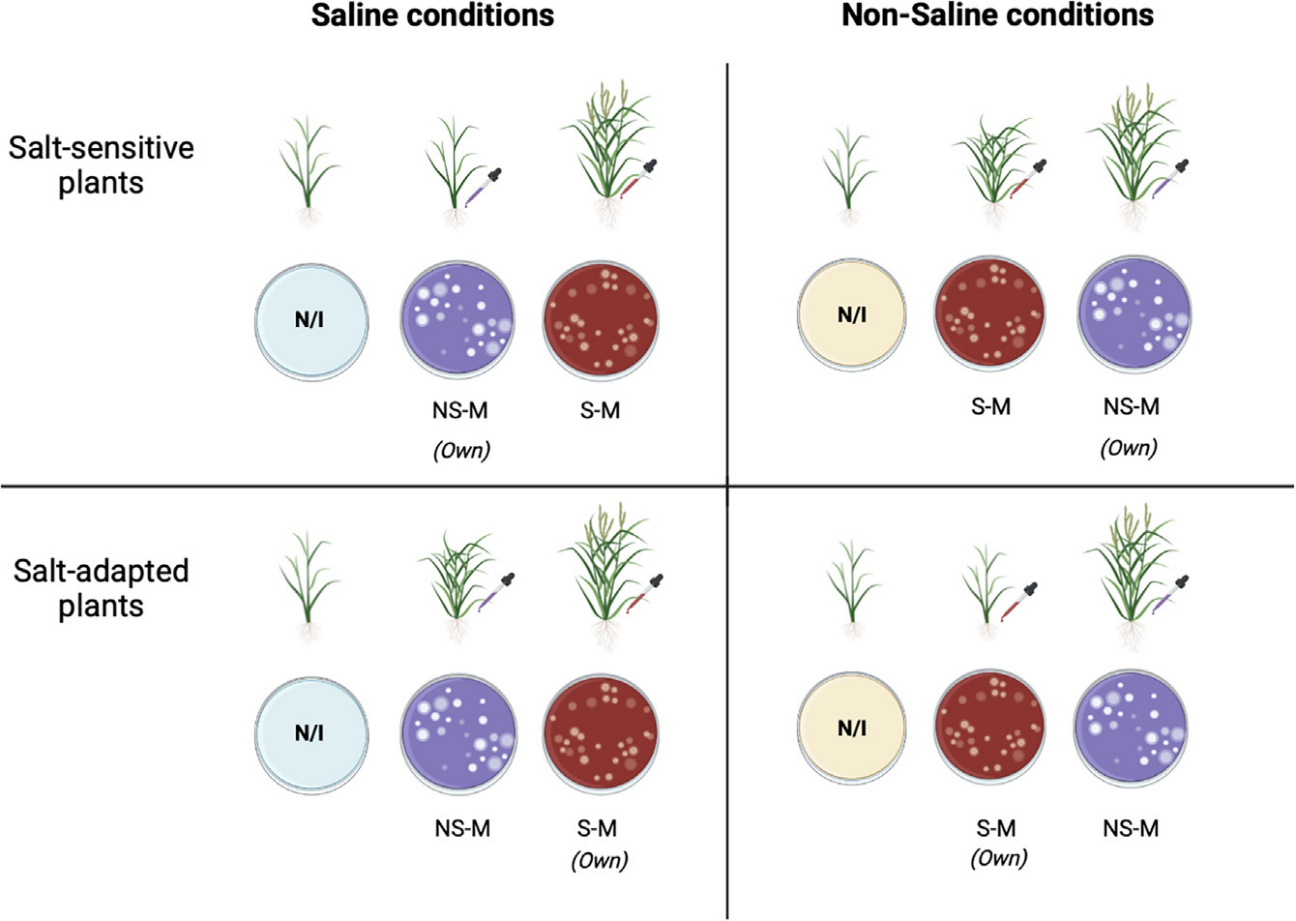
Experimental setup for a microbiome reciprocal transplant. Salt-adapted and salt-sensitive plants cultivated in sterile saline or sterile non-saline soil will be non-inoculated (N/I), inoculated with their own microbiome (saline microbiome [S–M] or non-saline microbiome [NS-M]), or inoculated with the opposite microbiome, in each of the four scenarios.

To understand evolved, adaptive soil-plant–microbiome associations, field transplant experiments that match the host and microbiome in an ecological setting are essential (Reed and Martiny, 2007). However, to assess the impact on plant performance of a particular microbiome, controlled environment reciprocal transplants using sterilized soil have been productive (Figure 2; e.g., Smith et al., 2018; Van Nuland et al., 2019). Although the composition of the microbiome cannot be easily manipulated in the field, applying these approaches can begin to tease apart the effects of the microbial composition of microbiomes from environmental parameters and, at the same time, allow the study of a single stressor such as soil salinity.

Saline soils are unique ecological niches inhabited by extremophilic microorganisms with specific adaptation strategies. For some years now, dedicated studies have aimed for the isolation and characterization of plant endophytes living in saline and other extreme environments (Otlewska et al., 2020). Around 350 species of the more than 1200 halophytes catalogued in the eHALOPH database are recorded as having associated microorganisms and mycorrhizal status (Santos et al., 2016). These represent a severely under-exploited reservoir for potential treatments against abiotic stresses affecting agriculture, including extreme temperatures, drought, salinity, or heavy metal stress (see Kumar and Verma, 2018). This halophytic root microbiome can positively influence the host through several routes: providing nutrients or favoring nutrient acquisition; modulating phytohormone levels, regulating antioxidant responses, synthesizing exopolysaccharides (EPS), maintaining plant defense against biotic stress, accumulating organic solutes such as proline and betaine, and increasing soil aggregation (Akyol et al., 2020).

Plant-growth-promoting rhizobacteria (PGPR) and mycorrhizae now have well-understood root colonizing capacities and some have can alleviate the inhibitory affect of salinity on plant growth (Zheng et al., 2021; Evelin et al., 2019). For example, Yasmin et al. (2020) found that *Pseudomonas pseudoalcaligenes* and *Bacillus subtilis* significantly improved the growth of soybean under salinity stress through the impact on a series of physiological regulatory processes mainly related to the activation of antioxidant defense system in order to reduced ROS overproduction. Parvin et al. (2020) concluded that specific arbuscular mycorrhizal fungi can promote salt tolerance and productivity in rice, likely by improving photosynthetic efficiency and K^+^/Na^+^ ratio, and restricting Na^+^ uptake and translocation. However, to date, these mechanisms have only been documented in a few cases, and their distribution in the whole microbial community of salinity-tolerant plants remains to be defined.

Core microbiomes are shared features of microbial communities that, because of their conservation, are inferred to have importance for host fitness, and therefore promise the potential to rationally manage plant microbiomes toward specific outcomes (Toju et al., 2018). Excellent earlier reviews have discussed a wide range of plant beneficial traits provided by diverse microbial groups under stress conditions (Friesen et al., 2011; de Zelicourt et al., 2013; Tkacz and Poole, 2015; Qin et al., 2016). In Table 2 we give an updated overview of relevant studies that have characterized saline-associated core microbiomes.

**Table 2:** Studies that have characterized saline-associated core microbiomes.

Environmental origin	Plant species	Microbiome type	Sampling	Comparison strategy	Most abundant taxa	Tested for salinity tolerance in host or non-host species:	Reference
Coastal habitats (high salinity) of Taiwan	*Miscanthus sinensis*	rhizosphere and endosphere compartments	20 samples: five sites, two specimens, two compartments	dominant bacteria across all samples	endophytic *Agrobacterium*, *Amycolatopsis* (with ACC deaminase enzyme) and denitrifying bacteria		Huang et al., 2020
Coastal cliffs in the North Atlantic coast of Spain	*Festuca rubra pruinosa*	endophytic mycobiota of roots	105 samples: around 20 samples from five sites	dominant endophytic fungi across all samples	*Fusarium*, *Diaporthe*, Helotiales, *Drechslera*, *Slopeiomyces*, and *Penicillium*		Pereira et al., 2019
Coastal habitats (eastern China)	*Suaeda salsa*	bulk soil and root endosphere	18 samples: three sites, three specimens, two fractions	dominant bacteria and fungi across all samples	Proteobacteria (α and γ), *Microbulbifer*, *Pelagibius*, *Halomonas*, *Marinoscillum*, *Fulvivirga*, *Haloferula*, *Pelagicoccus*, *Marinobacter*	cucumber, rice	Yuan et al., 2016
Coastal habitats of San Juan Island Archipelago (WA)	*Leymus mollis*	fungal endophytes	200 plants collected from several beach habitats in two different years	isolation of culturable fungal endophytes	*Fusarium culmorum*	tomato, rice	Rodriguez et al., 2008
Coastal salt marshes on Dauphin Island (Alabama)	*S. alterniflora* and *Juncus roemerianus*	rhizosphere microbiome	194 samples: two plant species, five replicates, from 4/2015 to 10/2016	core microbiome of both species in different samples from the same salt marsh	Anaerolineaceae; Planctomycetes, Proteobacteria (α and δ), Chloroflexi		Mason et al., 2021
Coastal salt marshes in southern Spain	*Arthrocnemum macrostachyum*	Bulk soil	eight samples: two locations, two replicates, two seasons	dominant bacteria	Proteobacteria, Actinobacteria, Bacteroidetes, Gemmatimonadetes, Chloroflexi, Firmicutes, Acidobacteria		Camacho-Sanchez et al., 2020
Coastal salt marshes of Jiangsu Province (China)	*Limonium sinense*	bacterial communities of the roots, leaves, rhizosphere, and bulk soils	12 samples: one site, three specimens, four fractions	dominant bacteria across all samples	Actinobacteria (*Glutamicibacter*, *Streptomyces*, *Isoptericola*); Firmicutes (*Bacillus*, *Lysinibacillus*, *Staphylococcus*); Proteobacteria (*Pseudomonas*, *Serratia*, *Klebsiella*, *Neorhizobium*)	*Glutamicibacter halophytocola* strain KLBMP 5180 tested in *L. sinense* under 250 mM NaCl	Qin et al., 2018
Coastal saline fields of west Bengal (India)	*Oryza sativa*	root endophytic bacteria	six agroecological regions, three sites, three specimens	dominant endophytic bacteria across samples from the coastal saline zone	Firmicutes and Proteobacteria		Kunda et al., 2021
Hypersaline ecosystems of southern Tunisia	*Salicornia* spp.	rhizosphere and bulk soil	18 samples: three sites, three specimens, two fractions	dominant bacteria across all samples	*Halomonas*		Mapelli et al., 2013
Saline habitats of northeastern Pakistan	*Suaeda fruticosa*	Rhizosphere and phytoplane		halotolerant bacteria	*Gracilibacillus*, *Staphylococcus*, *Virgibacillus*, *Salinicoccus*, *Bacillus*, *Zhihengliuella*, *Brevibacterium*, *Oceanobacillus*, *Exiguobacterium*, *Pseudomonas*, *Arthrobacter*, and *Halomonas*	*Staphylococcus jettensis* F-11, *Zhihengliuella flava* F-9, *Bacillus megaterium* F-58, *S. jettensis* F-11 and *S. arlettae* F-71 tested in *Z. mays* under 200 mM NaCl	Aslam and Ali, 2018
Saline site: salt mine (Khewra, Pakistan)	*Salsola stocksiii and Atriplex amnicola*	rhizospheric soil		*Bacillus*-derived bacterial (halophilic, alkaliphilic, and mesophilic)	*Bacillus*, *Halobacillus*, *Virgibacillus*, *Brevibacillus*, *Paenibacillus*, *Tumebacillus*, and *Lysinibacillus*		Mukhtar et al., 2018
Saline sites (anthropogenic and naturally) of central Poland	*Salicornia europaea*	endophytes of roots and shoots	36 samples: two sites, two seasons, three plots, three replicates	dominant endophytes across all samples	Proteobacteria and Bacteroidetes dominated bacterial assemblages, and Ascomycetes were the most frequent fungi. A root core microbiome of the genus *Marinimicrobium* was identified		Furtado et al., 2019
Saline sites of central Argentina	Chenopodiaceae (*Allenrolfea patagonica*, *Atriplex argentina*, *Heterostachys ritteriana* and *Suaeda divaricta*)	AMF of rhyzospheric soil and roots	40 samples: two sites, five depth intervals, four species	AMF diversity	19 morphologically distinctive AMFs (more present: *Glomus magnicaule*, *Septoglomus aff. constrictum*, *G. brohultti*, and *Septoglomus aff*.)		Becerra et al., 2014
Salterns of Secovlje (Slovenia)	12 halophytic plants	AMF and/or dark septate endophytes of rhyzospheric soil and roots	eight sites, 12 species, different number of individuals	AMF and DSE identification and colonization levels	co-occurrence: *Glomus* sp. and *Diversispora* sp. clades		Sonjaket al., 2009
Experimental field station at Shenyang Agricultural University (China); soil adjusted to 2.5 g (NaCl) kg^−1^to mimic a moderate soil salinity level	*Sorghum bicolor*, *Arachis hypogaea*, and intercropping system	peanut rhizosphere (IP), sorghum rhizosphere (IS), and interspecific interaction zone (II)	18 soil samples: three sites, three replicates, two years	core microbiome of both species in the three zones	dominant bacterial phyla: Proteobacteria, Bacteroidota, and Acidobacteriota. Dominant fungal phyla: Ascomycota, Basidiomycota, and Mucoromycota		Shi et al., 2021
Experimental field station of Shihezi University (China)	*L. mollis* (dune grass)	arbuscular mycorrhizal fungal		*G. mosseae* isolate from saline soil vs. non-saline soil	*Glomus mosseae*	cotton	Tian et al., 2004
Experimental field station: saline soil from the Shandong Academy of Agricultural Sciences (China)	*Glycine soja*, *Sesbania cannabina* and nonlegume *Sorghum bicolor*	bulk soil, rhizosphere, and nodule microbiome	36 samples: three plant species, three specimens, four compartments	core microbiome in the four compartments of two legumes and dominant bacteria in the nonlegume	dominant bacteria belonged to Proteobacteria and *Ensifer* for legumes and *Bacillus* for *S. bicolor*		Zheng et al., 2020 and 2021
Deserts and dry lands of Mexico and southern California	cultivated and native *Agave* spp.	rhizosphere, phyllosphere, leaf and root endosphere, proximal and distal soil	252 samples: 72 from *Agave tequilana*, 72 from *Agave salmiana*, and 108 from *A. desert*	core microbiome of three Agave species from different locations	Increased abundance of Proteobacteria and decreased presence of Acidobacteria. Dominated by members of Ascomycota		Coleman-Derr et al., 2016

AMF, arbuscular mycorrhizal fungi.

From this body of work, we suggest that Proteobacteria, Firmicutes, Ascomycota, and Glomeromycota appear to be the most abundant and non-species-specific bacterial and fungal taxa present in plant microbiomes from saline environments (Table 2). Representatives from both phyla could mitigate salt stress by direct mechanisms involved in protecting the plants (ACC deaminase, EPS, phytohormone production) or by indirect mechanisms based on modification of the plant metabolome. Of late, there has been a growth in studies correlating metabolomic and transcriptomic data to understand the crosstalk between plants and microorganisms (e.g., Wu et al., 2020; Salas-González et al., 2021; Rane et al., 2022). However, there is little information on expression changes in response to fluctuating abiotic stresses in the plant-microbiome-defined transcriptome. Dedicated metagenomic studies over time in natural conditions are required to fully understand these interactions. Together, this work will offer plant breeders the power to select the best cultivar-inoculum pairs in order to optimize resilience and yield of crops in the face of increasing climate volatility.

## A view toward future progress

Here we have focused on fascinating recent empirical examples of plant adaptation to extreme environments, highlighting both evolutionary and molecular mechanisms. We emphasized benchmark studies of ecologically adaptive salt tolerance in plants, highlighting the now quite clear interplay between salinity adaptation and both increased ploidy and the microbiome.

The rapid development of genomics based on both large-scale and long-read data to test evolutionary hypotheses is increasingly providing nucleotide-level resolution of the molecular mechanisms of adaptive evolution. This holds even for complex hazards and highly polygenic polyploid adaptation events (Konečná et al., 2021), long restricted largely to theoretical work (Haldane, 1930; Barton, 2022). As evolutionary genomics using very high-density data (thousands of complete genomes in single datasets to powerfully target candidate adaptive mechanisms) is increasingly combined with detailed assessments of adaptive phenotypes, we will rapidly identify adaptive mechanisms across plant diversity. A good choice for studies of molecular convergence in salt-adapted species would be the sequencing of diverse species that otherwise share the same niche and the same selective pressures (including the same host range), as has been attempted with various woody plants at the land-sea interface (He et al., 2020). Given also the pervasive role of structural genomic variation underlying adaptive evolution to edaphic stressors (most often through expression changes of transporters; see Baxter et al., 2008; Hanikenne et al., 2013; Busoms et al., 2018, 2021), we also underscore that long-read-based graphical pangenomics will play a key role in detecting these SVs in future studies of salinity tolerance. Already these studies are commonplace in major crops (Zhou et al., 2019, 2022a, 2022b; Liu et al., 2020a; Alonge et al., 2020; Song et al., 2020; Cai et al., 2021; Hämälä et al., 2021), and we foresee their application to studies of natural adaptive variation in the very near future.

However, the best future studies will not just employ high-throughput long-read pangenomics to probe the genomic basis of adaptation. They will naturally be explicitly interdisciplinary, combining innovations in functional phenomics, imaging, ionomics, and remote sensing with genomics. For example, to capture the finest-scale variability in phenotypic data of an entire region in high density and high throughput, automated drones will be used, capable of operating over extended time periods and over large areas, along with sensor loggers to monitor air humidity and soil moisture (Zribi et al., 2012). Broader adoption of such tools will greatly enhance our ability to understand and correlate environmental variation, which, for soil parameters, can shift within only a few meters, to genetic variation even within one site.

Such high-resolution studies have so far not been possible due to the expense of sequencing technologies and limitations in capturing environmental parameters, especially over time. It is now also obvious that microbiome characterization is required to ascertain soil health (Wilhelm et al., 2022), yielding datasets in which researchers can study microclimate associations with phenotypes, and to resolve the influence of individual abiotic components much more precisely. Also of great importance in such projects will be the use of machine learning algorithms, employed to handle large multidimensional genomic and phenotypic datasets (Lürig et al., 2021), through which predictions of gene-to-phenotype relationships will be greatly enhanced (Cheng et al., 2021; Jammer et al., 2022). All these innovations, the studies in natural conditions, and the integration of omic techniques considering not only the plants but also the microorganisms who cohabit with them will give a much clearer view of the fascinating and diverse natural mechanisms of salinity tolerance available in our ecosystems, thus allowing their adoption for the improvement of crops and our understanding of the fundamental mechanisms of evolutionary change.

## Funding

L.Y. gratefully acknowledges the support of a 10.13039/501100000275Leverhulme Trust Research Project Grant (RPG-2020-367).

## Author contributions

S.B., S.F., and L.Y. wrote the manuscript together.
